# Cyclin D1 + large B-cell lymphoma with altered CCND1 and BCL-6 rearrangements: a diagnostic challenge

**DOI:** 10.1186/s40364-019-0162-2

**Published:** 2019-06-03

**Authors:** Da Gao, Zach Liu

**Affiliations:** 1 Department of hematology and oncology, The affiliated hospital of Inner Mongolia Medical University, Hohhot, China; 2 Department of Pathology Rutgers University, New Brunswick, NJ USA

**Keywords:** DLBCL, Cyclin D1, CCND1, BCL-6, MCL

## Abstract

**Background:**

A subset of diffuse large B-cell lymphoma may show aberrant cyclin D1 expression, which may be confused with blastoid mantle cell lymphoma. These cases usually lack of CCND1 gene rearrangement. Duplication of CCND1 gene was attributed to some of the cases with cyclin D1 expression. The mechanism of overexpression of CCND1 in other cases was not well documented.

**Case presentation:**

We report a case of diffuse large B-cell lymphoma with cyclin D1 expression. The underlying mechanism for cyclin D1 expression was due to an abnormal gene rearrangement involving BCL-6 and CCND1, which was different from most reported cases. Rare cases with similar genetic profile were reported and were classified as diffuse large B-cell lymphoma.

**Conclusion:**

The phenotype and genetic abnormalities of DLBCL with cyclin D1 overexpression can be complex and may be difficult to differentiate from blastoid and pleomorphic variants of mantle cell lymphoma.

## Background

Diffuse large B-cell lymphoma, NOS (DLBCL), is a heterogeneous group of lymphomas with many morphologic and immunophenotypic variants and molecular subtypes. It can be subclassified into germinal center B-cell origin (GCB) and activated B-cells origin (ABC) based on its gene expression profile [1]. The GCB and ABC subtypes of DLBCL can be predicted using a panel of only 3 immunohistochemical stains (CD10, bcl-6, and MUM1). Compared with the cDNA microarray, this immunostaining panel reproduced the gene expression results in 71% of GCB and 88% of non-GCB cases and predicted for survival in a similar manner [2, 3]. BCL-6 gene rearrangement is the most frequent gene rearrangement in DLBCL and was reported in 30–35% of cases. Double-hit lymphoma is a subgroup of DLBCL with MYC rearrangement combined with BCL-2 and/or BCL-6 gene rearrangement, which has an aggressive course and poor prognosis [4]. More biomarkers are being evaluated for lymphoma diagnosis [5].

**Fig. 1:**
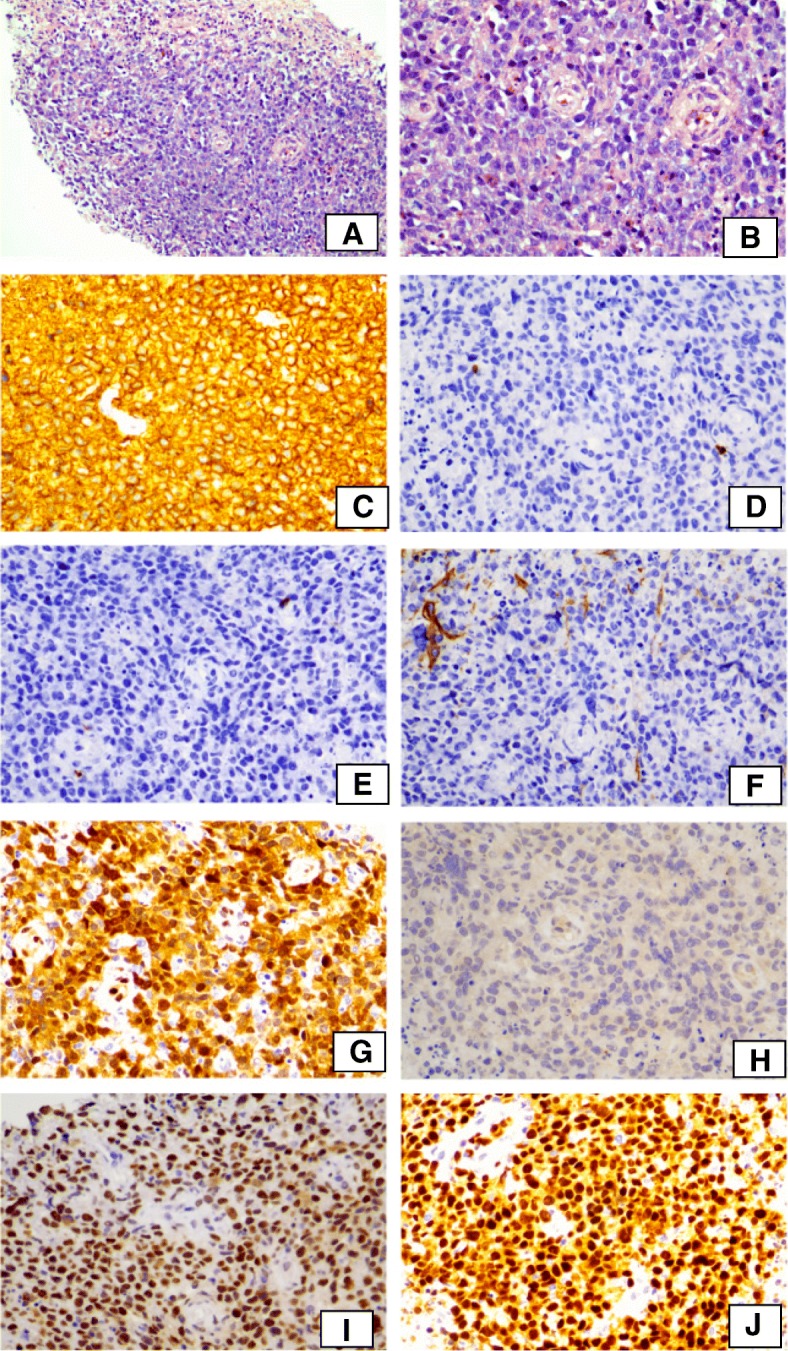
Lymphoma morphology and IHC results. **a** and **b** H&E, **c** CD20, **d** CD3, **e** CD5, **f** CD10, **g** CCND1, **h** SOX11, **i** BCL-1, and **j** MUM-1

**Fig. 2:**
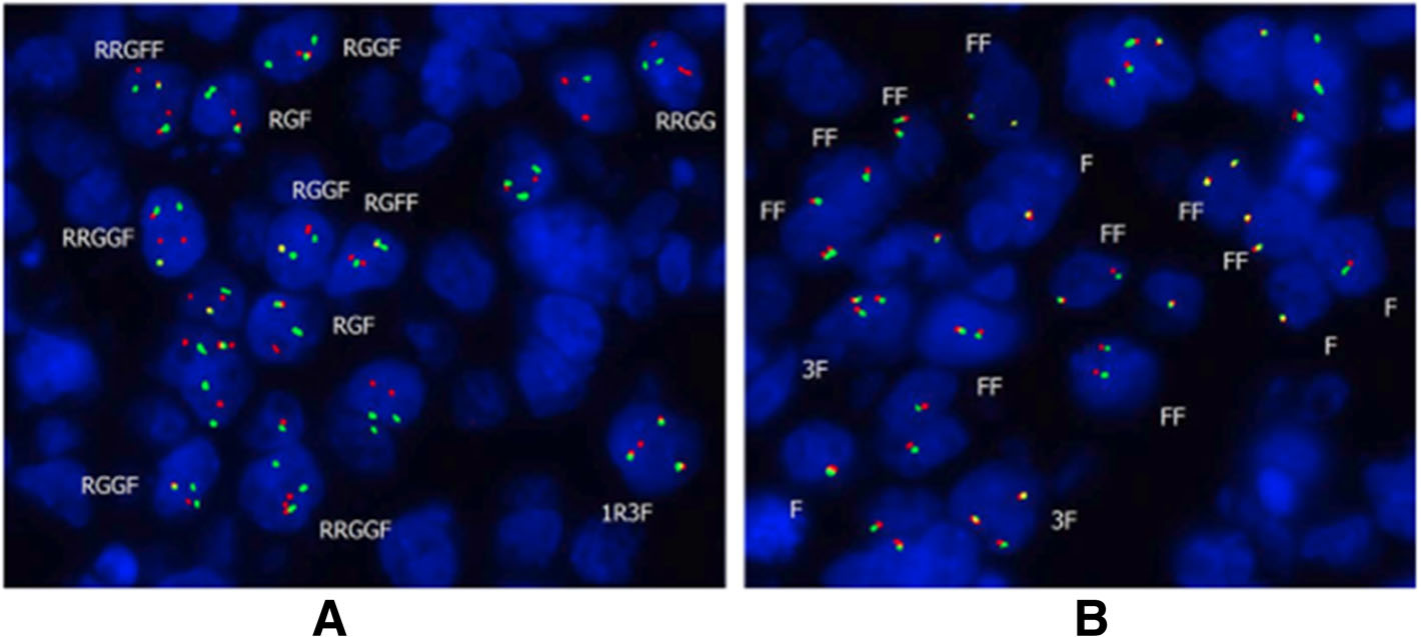
FISH results. **a** CCND1 rearrangement. **b** BCL-6 rearrangement

t(11;14)(13;q23) translocation between IgH and CCND1 is the hallmark of mantle cell lymphoma (MCL). cyclin D1 expression due to t (11;14, 13;q23) translocation between IgH and CCND1 is present in > 95% of cases including the minority of CD5 negative MCL. Variant CCND1 translocations do exist. In cases of cyclin D1 negative and lack of t (11;14)(q13;q23) MCL, cyclin D2 or cyclin D3 is highly expressed [6]. SOX11 staining is very valuable marker for diagnosis of MCL. Sox11 is positive in > 90% of MCL, including cyclin D1-negative and blastoid cases. Aberrant phenotypes have been described including absence of CD5, expression of CD10 or BCL-6 [7, 8], which may also be a pitfall for diagnosis.

Typically, the DLBCL can be distinguished from the blastoid or pleomorphic variant of MCL by the absence of CCND1/IgH translocation and lack of cyclin D1 and SOX11expression [9–11]. However, rare DLBCLs may show cyclin D1 expression in absence of t (11,14)(q13;q23) or SOX11 expression. These cases can raise a diagnostic challenge and misdiagnosed as MCL. Duplication of CCND1 gene was attributed to some of the cases with CCND1 expression. The mechanism of overexpression of CCND1 in other cases was not well documented. Herein, we report a case of DLBCL with cyclin D1 expression and unusual genetic rearrangement involving CCND1 and BCL-6. We also focus on the value of SOX11 in the differential diagnosis of mantle cell lymphoma and CCND1+ DLBCL.

## Case presentation

This was a 76-year-old man who was admitted to the hospital due to abnormal liver function tests and septic shock associated with systemic bacterial infection. Further workup during hospital stay revealed multifocal lymphadenopathy including cervical and inguinal regions. Ultrasound guided biopsy was performed from an inguinal lymph node. The patient was discharged after treatment of infection, improving cardiovascular symptoms and liver function. The patient electively refused treatment for lymphoma.

### Histology and immunohistochemistry

The biopsy showed diffuse large lymphoid cell infiltration with necrosis without recognizable follicular architecture. The lymphocytes showed moderate amount of cytoplasm and round nuclei with prominent nucleoli.

Immunohistochemical stains (IHC) with the following antibodies were performed following standard IHC protocol on Leica Bond Max stainer. The following antibodies from DAKO were used for staining: CD20, PAX5, CD3, CD5, CD10, CD23, CCND1, BCl-2, BCL-6, MUM1, SOX11 and Ki67.

IHC stains showed that the tumor cells were positive for CD20, cyclin D1, BCL6 and MUM-1. There was no CD5 or CD10 expression. SOX11 was negative (Fig. 1). EBER was negative. Proliferation index by ki67 was 80%.

### FISH

Interphase fluorescence in situ hybridization (FISH) was performed in the cytogenetic laboratory at Department of Pathology, Duke University Health System. Briefly, 4um sections were cut and de-paraffinized, FISH for CCDN1/IgH was performed using dual color, dual fusion probe from Abbott Molecular. This probe targets the CCND1 locus at 11q13 and the IgH locus at 14q23 for detection of the fusion gen associated with the translocation of 11;14. FISH for BCL-2, BCL-6 and myc were also performed using the probes from Abbott molecular.

Abnormal hybridization patterns with at least 2 fusion signals were overserved in 23/100 (23%) of the nuclei examined. The predominant abnormal signal pattern, presented in 13 cells, was consistent with two CCND1, one IgH and two fusion signals. Additional abnormal signal patterns with a single fusion signal were also observed. Gain and loss of signals, likely related to the truncation of nuclei in paraffin sections, was also observed.

39–52% of interphase nuclei examined showed an atypical pattern for the BCL6 locus consistent with either atypical rearrangement or partial deletion of the locus, and 3 signals for the IGH locus, consistent with CCND1/IGH fusion. No evidence of MYC (8q24.2) rearrangement or IGH/BCL2 fusion was observed. Taken together all the results from histology and molecular studies, this case was diagnosed to be diffuse large cell lymphoma (DLBCL) with BCL-1 expression (Fig. 2).

## Discussion and literature review

We described a case of DLBCL with BCL-1 expression. The phenotype of lymphoma cells was CD5-, CD10-, cyclin D1+, SOX11-, BCL2-, BCL6+ and MUM1+. In contrast to most of reported cases without rearrangement of t (11;14), our case showed gene rearrangement partially involved t (11;14). The predominant abnormal signal pattern for t (11;14) by FISH, presented in 13 cells, was consistent with two CCND1, one IgH and two fusion signals. 39–52% of interphase nuclei examined showed an atypical pattern for the BCL6 locus consistent with either atypical rearrangement or partial deletion of the locus. Evidence of MYC (8q24.2) rearrangement or IGH/BCL2 fusion was observed. The diagnosis was made based on typical DLBCL morphology, lack of SOX11 expression, concurrent BCL-6 rearrangement, lack of typical genetic changes of CCND1/IgH translocation. We believe that this case is best classified as DLBCL, rather than a blastoid variant MCL or a double hit MCL.

Two similar cases have been reported [6]. While the first biopsy specimen of case 1 was positive for BCL6 rearrangement and negative for CCND1 rearrangement on the initial biopsy, the subsequent biopsy specimen was positive for both IGH-CCND1 and BCL-6 gene rearrangements. The lymphoma cells were clonally related based on IgH rearrangement study. Several reciprocal translocations were identified on the second case, including t (3;22), t (4; 12), t (8;13), and t (11;14) translocations. Interphase FISH performed on the paraffin sections identified BCL6 gene rearrangement in 57.5% of cells analyzed and CCND1 rearrangement in 41% (5.5% double fusions and 35.5% single fusion) of cells. The presence of BCL-6 rearrangement, despite of concurrent CCND1 rearrangement, supported the diagnosis of DLBCL as authors stated. Phenotypically both cases were positive for MUM-1 and BCL-6, negative for CD5 or CD10. BCL-1 was positive and SOX11 was negative for both cases. The authors concluded these cases were DLBCL, not blastoid variant MCL. Our case together with these two cases are unique in a way that they both harbor BCL6 rearrangement in addition to altered IGH-CCND1, providing additional evidence for the existence of cyclin D1–positive DLBCL with abnormal IGH-CCND1 [7].

CCND1 gene rearrangements were also reported in another case report by Juskevicius et al. [12], in which one of the two cases showed t (11,14) and BCL6 arrangement, and the gene copy number changes detected by CGH more resembling those from DLBCL rather than MCL; the second case showed complex translocations. The break-apart probe for CCND1 showed split signals in the tumor cells, but the CCND1/IGH probe failed to detect gene fusions. Chromosome analysis using spectral karyotyping on the cells from the bone marrow biopsy of case 2 revealed a complex translocation t (4;11;14)(q22;q13;q32), and metaphase-FISH showed that a major fragment of chromosome 14 bearing the IgH locus was translocated to the long arm of chromosome 4, masking the translocation for conventional interphase-FISH. Both cases were negative for SOX11. More importantly, from the clinical perspective, the course of the first patient did not favor a diagnosis of MCL. This elderly patient achieved complete remission after R-CHOP treatment and was still alive without evidence of recurrence 6 years after diagnosis. The disease course of the second patient was somewhat different in that he was diagnosed with a more widespread lymphoma involving the bone marrow, but he was in complete remission 1 year and 9 months after high-dose chemotherapy with autologous stem cell rescue.

It is debatable how to classify these cases of large cell lymphoma with involvement of CCND1 gene rearrangement like our case and the cases from Al-Kawaaz et al. It is possible that there is a grey zone between the blastoid variant of MCL and DLBCL as speculated by Juskevicius et al. However, based on the overall clinical and genetic findings, DLBCL was favored by both Al-Kawaaz and Juskevicius’s studies.

Question was also raised whether this case was a double hit lymphoma. Double hit lymphoma (DHL) is defined as a B-cell lymphoma with a MYC translocation combined with an additional translocation involving other known oncogenes, such as BCL-2 and BCL-6 [13]. DHL has been well studied and has an aggressive clinical course and poor prognosis [4, 14]. The hallmark of cytogenetic event in MCL is the t (11;14)(q13;q32) between the IGH and CCND1 genes [15–17]. The double hit MCL is superimposed by additional myc rearrangement. Approximately 5% of all mantle cell lymphomas (MCLs) in the database were CCND1+/MYC+ DH cases [13]. Activation of MYC may bring the cells in an advantageous metabolic state, allowing cells to progress further. Acquisition of a MYC translocation is associated with a dramatic morphologic change in MCL [13]. A case reported by Kallen et al. illustrated this point very well. In this report, both BM and PB karyotypes had t (11;14) along with several secondary aberrations. The FISH studies on cultured peripheral blood cells showed CCND1-IGH fusion signals t (11;14) in 74.3% and monosomy 13 in 79.7% of nuclei examined. FISH performed on FFPE sections of the bone marrow biopsy showed multiple copies (∼3–5) of the MYC gene locus at 8q24 in 73.7% of nuclei examined [18]. Our case showed low number of CCND1/IgH translocation (23%) with unconventional pattern. 39–52% of cells showed BCL-6 with unconventional rearrangement. Therefore, it is unlikely that our case was a MCL with subsequent double hit.

Several large series and case reports have been published regarding cyclinD1/BCL-1+ DLBCL. The prevalence is about 1–2% depend on the study [17, 19, 20], while Ehinger et al. reported 4.3% (10 out of 231) and Velar et al. reported 15% (10 out of 66). Ok et al. went even further and reviewed 1435 cases from the DLBCL Ritoximab-CHOP consortium program, the prevalence was 2.1% [7]. Most of these reported cases were negative for t (11;14) translocation. The overexpression of cyclin D1 was attributed to altered/increased CCND1 gene copy number in a subset of the cases [7, 17, 21]. The mechanism of overexpression of cyclin D1 in other cases was not well documented. Compared with patients who had cyclin D1-negative DLBCL, men were more commonly affected with cyclin D1-positive DLBCL, and they were significantly younger. There were no other significant differences in clinical presentation, pathologic features, overall survival, or progression-free survival between these two subgroups of patients with DLBCL (6). Recent publication of Cyclin D1-positive mediastinal large B-cell lymphoma with copy number gains of CCND1 gene by Chan further supported the above observation [22].

Initial reported cases of DLBCL with cyclin D1 expression were thought as non-GC phenotype. However, the study from Ok et al. showed only 56.7% were non-GC cell origin. The non-GC type was typically positive for MUM-1 and the GC type was typically CD10+ and BCL6+. CD5 positive DLBCL cases were rare. Our case was CD5-, CD10-, MUM-1+, consistent with a no GC type. Regardless, both type of GC or non-GC DLBCL should be negative for SOX11.

Because of cyclin D1 expression, MCL is inevitably a differential diagnosis. MCL is a distinctive lymphoma type characterized by the presence of CCND1 translocation with overexpression of cyclin D1 and positive SOX11. MCL usually presents with advanced stage and rapid clinical progression. The diagnosis is in most instances uncomplicated, but cases with variant morphologies or immunophenotypes may cause diagnostic difficulties.

Unusual variants of MCL have been documented and reviewed by Fratoni in 2017 [23]. Morphologically, the classic variant, composed of the “classical” morphology, is detectable in about 90% of MCL cases, while the remaining approximately 10% are considered MCL variants, which include the small cell variant (chronic lymphocytic leukemia-like), the monocytoid-like variant, the MCL with plasmacytic differentiation, the pleomorphic variant and the blastoid variant. The latter two variants need to be differentiated from DLBCL especially in situation with aberrant phenotype.

Typically, the phenotype of MCL is CD5+, cyclin D1+ and SOX11, and negative for CD10, CD23, BCL-6 and MUM-1. Variations in immunophenotypes do occur. Lack of CD5 expression is well documented, as reported as early as 2002 [8]. Aberrant expression of CD10 and BCL6 in MCL has been reported. In Pizzi’s report, BCL6 and /or CD10 expression were documented in 15.8% (26 out of 165) of MCL; in particular BCL6 were detected in 10.3% (17 out of 165), and CD10 in 6.7%). two of 165 samples showed double positivity for CD10 and BCl6. MUM-1 was also detected in some of the cases [21]. While BCL6+/MUM1+ were seen in most (75%) cyclin D1–positive DLBCLs (13), the aberrant expression of BCL6 and MUM1 also appeared on classical, pleomorphic and blastoid variants of MCL.

The SOX11 gene, mapping at chromosome 2p25.3, encodes a transcription factor of 441 amino acids, which belongs to a family of approximately 20 genes characterized by the presence of a conserved DNA-binding high-mobility group domain. SOX11 emerged as a very useful marker for the diagnosis of MCL to distinguish from DLBC with cyclin D1 expression [19, 24–27]. Mozos et al. investigated protein expression by immunohistochemistry in a series of 54 cyclin D1-positive MCL, and 209 other lymphoid neoplasms, virtually all MCL were strongly positive for SOX11 (50/54, 93%), with a nuclear pattern. SOX11 was positive also in the 12 cyclin D1-negative cases of MCL, six cases of lymphoblastic lymphomas, two of eight cases of Burkitt’s lymphoma, and two of three T-prolymphocytic leukemias, but was negative in the remaining lymphoid neoplasms. Of note, more than half of the cyclin D1 negative cases (22/40) showed CCND2 translocation [28].

SOX11 plays an import role in the differential diagnosis of MCL. Recent studies on molecular pathogenesis in the development of MCL provided better understanding of CCND1 and SOX 11 expression. MCL can be categorized into two types based on the proposed cell origin. One type is abnormal naive cells, IgVH unmutated/minimally mutated, colonized in the mantle zones, these cells already have additional molecular genetic abnormalities, such as inactivating ATM mutations. This type of MCL frequently involves lymph nodes and extranodal tissue. They may progress to classical MCL which most frequently is SOX11+; ultimately, progression to blastoid or pleomorphic MCL may occur. The second category is MCL with somatic hypermutation, leading to SOX11- MCL that are more genetically stable for long periods of time and which preferentially involve peripheral blood, bone marrow (BM), and sometimes the spleen. These MCL, however, may undergo additional molecular/cytogenetic abnormalities, particularly TP53 abnormalities, leading to clinical and sometimes morphological progression [29].

For diagnosis purpose, SOX11 expression is primarily analyzed by immunohistochemistry (IHC). Molecular quantification of SOX11 in conjunction with immunoglobulin variable heavy chain gene mutation (IgVH) status may contribute to the identification of two forms of MCL (IgVH mutated and unmutated), may also be used for MRD quantification. Magne et al. described an RT-qPCR assay for SOX11 expression to better characterize MCL at diagnosis. By using appropriate housekeeping genes, the authors studied a cohort of B-cell malignancies to define an easy-to-use molecular tool for distinguishing MCL from other B lymphoproliferative disorders [30].

The few reported cases of DLBCL with CCND1 involvement were treated as DLBCL [31]. The outcomes were variable. The coordinate genetic signatures may be useful in providing a roadmap for an actionable DLBCL classification [32–34]. The DLBCL outcome-associated genetic signatures may guide the development of rational single-agent and combination therapies and or targeted therapy in the future, such as those emerging therapies for mantle cell lymphoma [35–38].

## Conclusion

The phenotype and genetic abnormalities of DLBCL with cyclin D1 overexpression can be complicated and may be difficult to differentiate from the blastoid and pleomorphic variants of MCL. Comprehensive IHC and molecular/genetic studies are essential for an accurate diagnosis. If a DLBCL with aberrant expression of cyclin D1 is detected, complete work-up including IHC for CD5, CD10, BCL6, MUM-1 and SOX11, and FISH for CCND1, BCL-2, BCL6, c-myc should be performed to better characterize the neoplastic process.

## Data Availability

The lymphoma diagnostic H&E slides, IHC slides and FISH report from Duke university are available.

## Abbreviations

DH: Double hit
DLBCL: Diffuse large B-cell lymphoma.
EBER: Epstein-Barr virus-encoded mRNA
EBV: Epstein-Barr virus
FISH: Fluorescence in-situ hybridization
GCB: Germinal center B-cell
MCL: Mantle cell lymphoma
R-CHOP: Rituximab plus cyclophosphamide, doxorubicin, vincristine and prednisolone: RT
SOX11: Sex-determining region Y-box 11
WHO: World Health Organization
